# “Swirl Sign”: A Case of Abdominal Pain After Roux-en-Y Gastric Bypass Surgery

**DOI:** 10.5811/cpcem.2018.3.37196

**Published:** 2018-07-12

**Authors:** Carlin Corsino, Hillary Harper, Vanessa Sieg

**Affiliations:** Carl R. Darnall Army Medical Center, Department of Emergency Medicine, Fort Hood, Texas

## CASE PRESENTATION

**Image 1:**
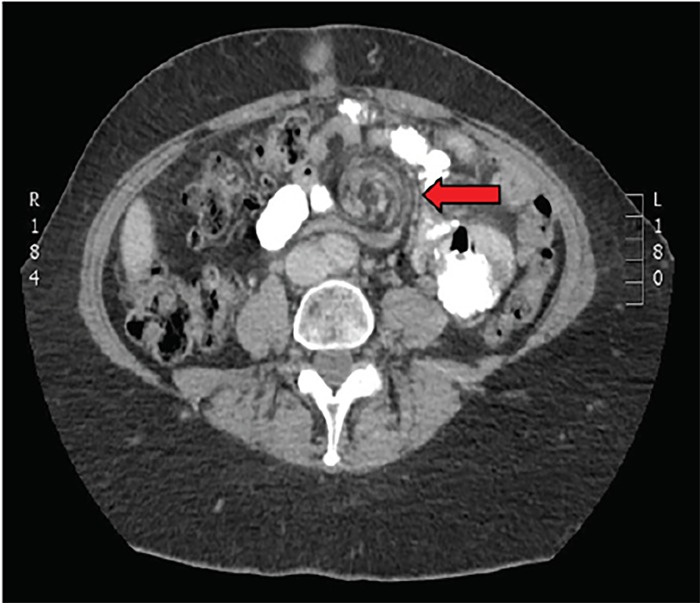
Axial computed tomography of the abdomen shows “swirl sign” indicative of internal hernia (red arrow).

**Image 2:**
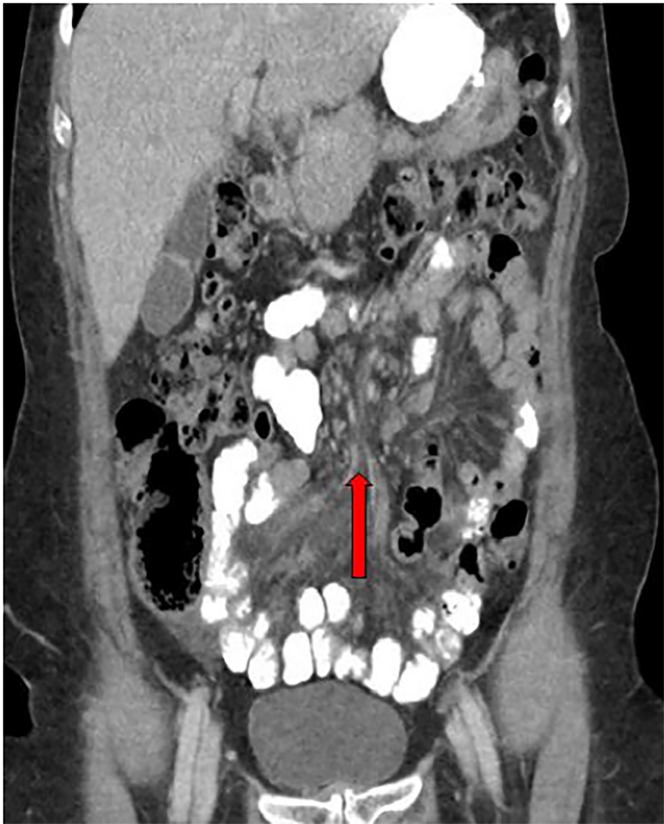
Coronal computed tomography of the abdomen and pelvis showing site of internal hernia (red arrow).

A 57-year-old female presented to the emergency department (ED) with periumbilical and left upper quadrant abdominal pain. The pain began abruptly 12 hours prior to presentation and was worsening. Her pain increased with supine position and was associated with nausea and vomiting. Her past medical history was significant for hypertension, gastroesophageal reflux disease and obesity. Prior to presentation in our ED, she underwent a laparoscopic Roux-en-Y procedure for weight loss 10 years prior at an outside hospital. On arrival, pertinent vitals included a heart rate of 115 beats per minute, 20 breaths per minute and blood pressure of 190/100 mmHg. Laboratory studies in the ED were significant for a leukocytosis (14.7 × 10^9^/L), and a lactate level of 5.4 mmol/L. The remainder of laboratory studies were normal. Computed tomography (CT) images were obtained (Images 1 and 2).

## DIAGNOSIS: INTERNAL HERNIA

Obesity is an epidemic in America, and bariatric surgery is becoming more common. Roux-en-Y procedure is the “gold standard” of bariatric surgery.^1^ It provides more overall weight loss than adjustable gastric band and more durable weight loss than sleeve gastrectomy.^1^,^2^ Complications of Roux-en-Y gastric bypass are categorized as early or late. Early complications include anastomotic or staple-line leak, hemorrhage and obstruction. Later complications can be difficult to differentiate from other more routine abdominal emergencies seen in the ED. Late complications include anastomotic stricture, marginal ulceration, fistula, nutritional deficiencies and bowel obstruction.^3^ Internal hernia can occur at any time after the procedure and has lifetime incidence of roughly 5%.^4^ Ironically, the potential space created by sudden, post-procedural weight loss is a risk factor for this complication.^5^

Internal hernias develop when bowel protrudes through iatrogenic defects in the mesentery. This is most common at the transverse mesocolon, Petersen’s space, or the meso/jejunojejunal anastamosis. Petersen’s space is a defect posterior to the Roux limb.^6^ Symptoms of internal hernia can be intermittent, vague and may mimic benign disease processes. This makes diagnosis of this uncommon yet life-threatening finding particularly difficult.

Diagnosis can be made by CT, where the “swirl sign” is sometimes seen (Images 1 and 2). If present it is 78–100% sensitive, and 80–90% specific for internal hernia.^7^ Even in the absence of swirl sign, patients should undergo exploratory surgery if suspicion of internal hernia is high based on clinical presentation, unexplained laboratory abnormalities that may suggest bowel ischemia, or imaging consistent with the stigmata of bowel obstruction. This patient underwent laparoscopic revision of her Roux-en-Y, and was discharged home after the procedure with no further complications.

Documented patient informed consent and/or Institutional Review Board approval has been obtained and filled for publication of this case report.

**CPC-EM Capsule**

What do we already know about this clinical entity?

Internal hernias are a post-operative complication of bariatric surgeries. Diagnosis can be made by computed tomography (CT) imaging showing a characteristic “swirl sign.”

What is the major impact of the images?

While the surgically altered abdomen may seem intimidating anatomically, there are tell-tale abnormalities that can be easily recognized on CT by the informed physician.

How might this improve emergency medicine practice?

We can be better advocates for bariatric surgery patients by knowing their potential post-operative complications and associated findings on imaging.
